# An Oxalate-Bridged Copper(II) Complex Combining Monodentate Benzoate, 2,2′-bipyridine and Aqua Ligands: Synthesis, Crystal Structure and Investigation of Magnetic Properties

**DOI:** 10.3390/molecules25081898

**Published:** 2020-04-20

**Authors:** Francielli Sousa Santana, Matteo Briganti, Rafael A. Allão Cassaro, Federico Totti, Ronny Rocha Ribeiro, David L. Hughes, Giovana Gioppo Nunes, Dayane Mey Reis

**Affiliations:** 1 Departamento de Química e Biologia, Universidade Tecnológica Federal do Paraná, Cidade Industrial, Curitiba 81280-340, Brazil; francielli.s.santana@ufpr.br; 2 Departamento de Química, Universidade Federal do Paraná, Centro Politécnico, Jardim das Américas, Curitiba 81531-980, Brazil; matteo.briganti@unifi.it (M.B.); ronny.ribeiro@ufpr.br (R.R.R.); nunesgg@ufpr.br (G.G.N.); 3 Instituto de Química, Universidade Federal do Rio de Janeiro, Rio de Janeiro 21941-909, Brazil; allao.cassaro@iq.ufrj.br; 4 Dipartimento di Chimica “U. Schiff” and UdR INSTM Università degli Studi di Firenze, Via della Lastruccia 3-13, 50019 Sesto Fiorentino, Italy; federico.totti@unifi.it; 5 School of Chemistry, University of East Anglia, Norwich NR4 7TJ, UK; D.L.Hughes@uea.ac.uk

**Keywords:** dinuclear copper(II), ferromagnetic interaction, magnetic properties, noncovalent interaction

## Abstract

A dinuclear copper(II) complex of formula [{Cu(*bipy*)(*bzt*)(OH_2_)}_2_(μ-*ox*)] (**1**) (where *bipy* = 2,2′-bipyridine, *bzt* = benzoate and *ox* = oxalate) was synthesised and characterised by diffractometric (powder and single-crystal XRD) and thermogravimetric (TG/DTG) analyses, spectroscopic techniques (IR, Raman, electron paramagnetic resonance spectroscopy (EPR) and electronic spectroscopy), magnetic measurements and density functional theory (DFT) calculations. The analysis of the crystal structure revealed that the oxalate ligand is in bis(bidentate) coordination mode between two copper(II) centres. The other four positions of the coordination environment of the copper(II) ion are occupied by one water molecule, a bidentate *bipy* and a monodentate *bzt* ligand. An inversion centre located on the ox ligand generates the other half of the dinuclear complex. Intermolecular hydrogen bonds and π-π interactions are responsible for the organisation of the molecules in the solid state. Molar magnetic susceptibility and field dependence magnetisation studies evidenced a weak intramolecular–ferromagnetic interaction (*J* = +2.9 cm^−1^) between the metal ions. The sign and magnitude of the calculated *J* value by density functional theory (DFT) are in agreement with the experimental data.

## 1. Introduction

Copper(II) complexes are interesting in coordination chemistry because of their vast applicability for bioinorganic purposes and synthesis of metallodrugs [1,2], catalysis [3,4] and magnetism [5,6]. Concerning molecular magnetism, it is known that the magnetic interaction between two or more copper(II) centres is strongly dependent on the nature of the bridging ligand that works like a magnetic exchange pathway [7,8]. Among these ligands, the oxalate ion, ox^2−^, for example, is well known for its capability to create adequate magnetic exchange pathways for ferro- and antiferromagnetic interactions in oligonuclear copper(II) compounds [9,10,11]. The combination of oxalate and copper(II) ions leads to a large structural variety including different nuclearities such as mononuclear [12,13], dinuclear [14,15], trinuclear [16,17], tetranuclear [17,18] and hexanuclear species [19,20] and coordination polymers [14,21]. This class of oxalate-bridged compounds is noteworthy in magnetic applications, as it may comprise many other transition metal ions such as Mn^II^, Fe^II/III^, Co^II^, Ni^II^, Cr^II/III^, V^IV^ and Ru^II^ [22,23,24,25].

The dinuclear copper(II) complexes, which contain only one unpaired electron per metallic ion, are the most straightforward systems to investigate the through-ligand electron exchange mechanism from experimental and theoretical perspectives [26,27,28]. It is well known that the nature and strength of the magnetic interactions in dinuclear complexes including simple inorganic and extended organic bridging ligands such as hydroxo, azide, aromatic dicarboxylate, diamine, oxalate and related derivatives are highly dependent on the nature of the chelating terminal blocking ligands, which prevent complex polymerisation [5]. Furthermore, the variation in the spatial arrangement of terminal ligands may change the orbital overlap angle between the copper(II) and the bridging ligand, leading to different types of magnetic interactions [29,30].

Regarding oxalate-bridged copper(II) compounds, the ferromagnetic or antiferromagnetic behaviour is determined by the overlap between the two magnetic orbitals. Poor overlap through oxalate leads to a weak antiferromagnetic interaction. On the other hand, if the overlap is zero, one could expect a ferromagnetic coupling [8]. Steric hindrance arising from the volume of the ancillary ligands leads to different orientations of the magnetic orbitals concerning the bridge plane, hence generating a different magnitude and nature of the magnetic interaction among the copper(II) centres [17].

Although a variety of oxalate (ox^2−^)-bridged copper(II) complexes can be found in the literature, just a few contain more than one bulky ligand besides the oxalate bridge. A detailed search carried out in the Cambridge Structural Database (CSD) [31] for oxalate-bridged copper(II) complexes with ox^2–^ in bis-bidentate coordination mode displayed a total of 423 results, of which 86 correspond to dinuclear complexes that possess two additional ligands besides ox^2–^. Out of 86 structures, 66 contain pentacoordinate metal ions bound to only one type of organic ligand such as pyridine [1], 2,2′-bipyridine [11,32], phenanthroline [33,34], ethylenediamine [35], pyrazole [36,37] and imidazole [38] and their derivatives, the coordination sphere being completed by simple inorganic ions such as nitrate [39], chloride [33], hydroxide [40] and perchlorate [1], or by solvent molecules such as water [6], methanol [34], dimethylformamide [41], tetrahydrofuran [42] or acetonitrile [43]. In the 20 remaining structures, all of which have been reported more recently, the metal ions are hexacoordinate and the chemical composition of the complexes, regarding the presence of at least one organic ligand, solvent molecules and simple inorganic ions, is similar to that observed for the pentacoordinate species [8,11,14,15,44,45]. Exceptions are found when tetradentate ligands direct hexa-coordination, decreasing the need for other molecular entities to complete the coordination sphere of the copper(II) ion [46,47]. Therefore, such systems show high structural and electronic versatility, which can be modulated by an appropriate choice of ligands other than the ox^2−^ bridge.

Herein the synthesis of a new heteroleptic oxalate-bridged copper(II) dinuclear complex [{Cu(*bipy*)(*bzt*)(OH_2_)}_2_(μ-*ox*)] (**1**) (where *bipy* = 2,2′-bipyridine, *bzt* = benzoate and *ox* = oxalate) is reported, in which the coordination environment of the metal ion is described as distorted octahedral with the unusual coordination of two relatively bulky ligands, a bidentate 2,2′-bipyridine and a monodentate benzoate, in addition to one water molecule. Structural correlations on how the orientation of the magnetic orbitals of the Cu^II^ ions is affected by the crystal field in **1** and the nature of magnetic interactions were investigated. We have also carried out theoretical calculations on the electronic structure and the electron paramagnetic resonance spectroscopy (EPR) spectra to bring light into its magnetic properties.

## 2. Results and Discussion

**Scheme 1:**
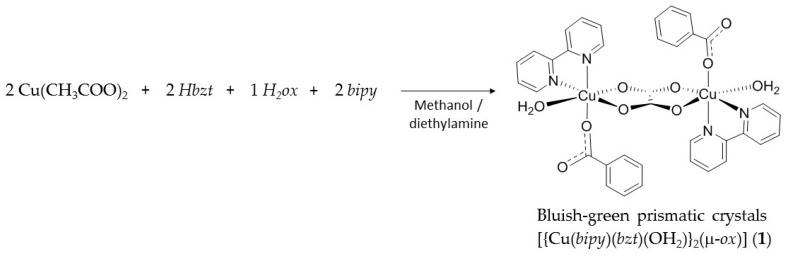
Synthetic methodology carried out between Cu(CH_3_COO)_2_·H_2_O, H*bzt*, H_2_*ox*·2H_2_O and *bipy* to produce complex **1**. Where *bipy* = 2,2′-bipyridine, *bzt* = benzoate and *ox* = oxalate.

The reaction between copper(II) acetate monohydrate, benzoic acid, 2,2′-bipyridine and oxalic acid dihydrate in methanol gave the bluish-green prismatic shape crystals of [{Cu(*bipy*)(*bzt*)(OH_2_)}_2_(μ-*ox*)] (**1**) in high yield, ca 90%, from a reproducible synthetic methodology (Scheme 1). Powder X-ray diffraction (PXRD) analysis revealed good correspondence between the simulated and experimental diffraction patterns (Figure S1 and Table S1).

### 2.1. Crystal Structure

**Figure 1:**
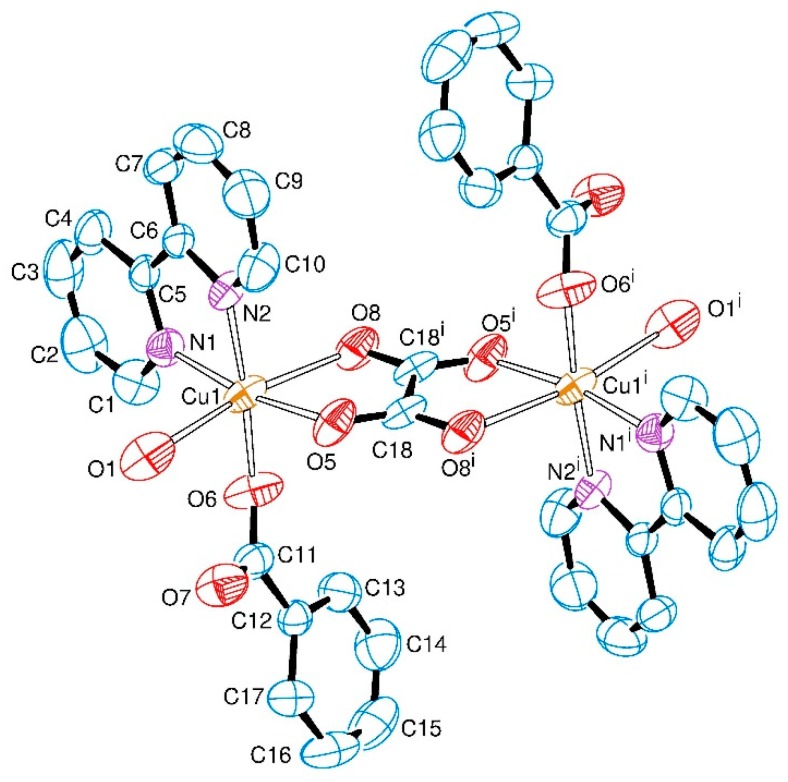
ORTEP representation of the [{Cu(*bipy*)(*bzt*)(OH_2_)}_2_(μ-*ox*)] (**1**), indicating the atom numbering scheme. The hydrogen atoms were omitted for clarity. Thermal ellipsoids are drawn at the 50% probability level. Symmetry code (i): 1 − x, 1 − y, 1 − z.

Complex **1**, Figure 1, was obtained as single-crystals that belong to the centrosymmetric space group *P*2_1_/*n*. The asymmetric unit consists of a copper(II) centre coordinated to one of each ligand molecule or ion, namely 2,2′-bipyridine, water, benzoate and one half of the oxalate bridge (the asymmetric term refers to the different C-O bond lengths in the asymmetric unit).

The equatorial positions on each copper(II) ion are occupied by two nitrogen atoms from the 2,2′-bipyridine ligand (N1, N2), one oxygen atom from the bridging oxalate group (O5) and one oxygen atom from the monodentate benzoate ligand (O6). The apical positions are occupied by the oxygen atom from the water molecule (O1) and the second oxygen atom from the bridging oxalate group (O8). There is an inversion centre lying in the middle of the C18–C18^i^ bond of the oxalate ligand that bridges the two copper(II) ions in the usual bis(bidentate) mode, giving rise to the neutral dimeric entity of [{Cu(*bipy*)(*bzt*)(OH_2_)}_2_(μ-*ox*)] as shown in Figure 1.

The coordination environment of the metal ion can be described as distorted octahedral with significant deviations exemplified by the O5–Cu1–O8 and O8–Cu1–O1 angles of 76.62(7)° and 170.00(7)°, respectively. The oxalate bridge is asymmetrically coordinated to the copper(II) centres, as indicated by the Cu1–O5 and Cu1–O8 bond lengths of 1.9732(19) and 2.378(2) Å, respectively. Equatorial Cu–N (Cu–N1 and Cu–N2) and Cu–O (Cu1–O5 and Cu–O6) distances average to 2.007(2) Å and 1.9578(19) Å, respectively, and are in good agreement with the corresponding bond lengths reported in the literature for other dimeric oxalate-bridged copper(II) complexes with the 2,2′-bipyridine ligand [6,11,32]. Due to the Jahn–Teller effect, the Cu1–O8 and the Cu1–O1 bonds are elongated (2.378(2) Å and 2.426(3) Å) in comparison to the other bond lengths in the coordination sphere of the metal in **1** (Table 1). These values are in good agreement with related tetragonally-distorted copper(II) coordination compounds that contain benzoate and/or an oxalate-bridging ligand [11,48]. The oxalate bite angle at the metal ion of 76.6° is also in accordance with other copper(II) complexes containing asymmetric oxalate bridges [8,49]. The selected geometric parameters of **1** are shown in Table 1.

The monodentate, rather than bidentate, coordination mode of the benzoate ion to the copper(II) centre is a remarkable feature of **1**. It may be explained by its shorter Cu-N bonds lengths (ca 2.0 Å) than the equivalent ones in analogous manganese(II) [50,51] or cobalt(II) [52,53] complexes, of ca 2.2–2.3 Å and 2.1–2.2 Å, respectively. These short bonds lengths may make it difficult to accommodate bulky ligands around the copper(II) centre. The monodentate binding of *bzt^−^* is interesting since it leads to hexacoordination, a surprising result for oxalate-bridged copper(II) systems according to an extensive CSD database search. As already discussed in the Introduction, the metal ion is usually pentacoordinate when the oxalate-bridged copper(II) ions possess two additional ligands besides ox^2^*^−^*. Therefore, complex **1** is one of the fairly rare oxalate-bridged copper(II) complexes in octahedral coordination involving more than one bulky ligand [44,54,55,56].

**Figure 2:**
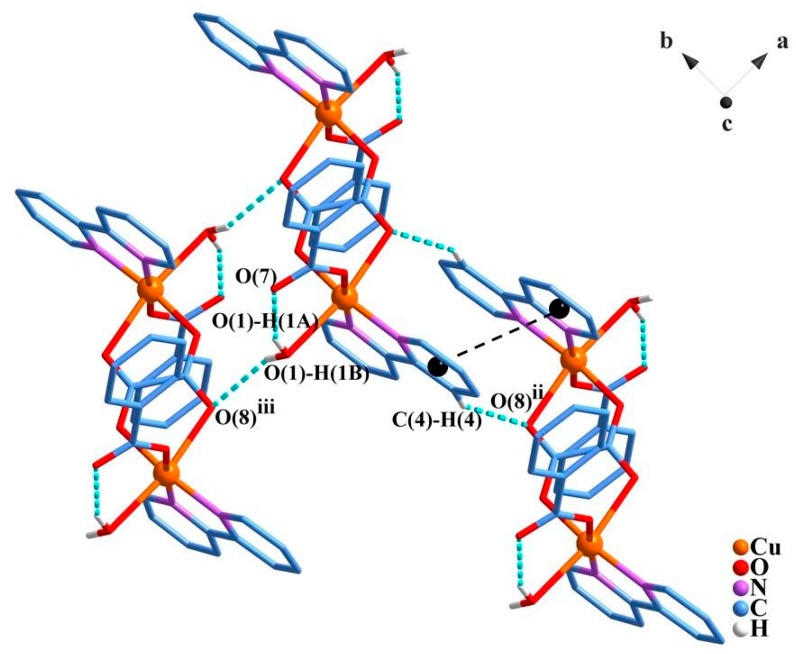
Representation of the hydrogen bonds (dotted blue lines) and π-π stacking (dotted black lines) between the *bipy* ligands with centroids represented by black spheres. The hydrogen atoms which do not perform hydrogen bonds were omitted for clarity. Symmetry codes: (ii) –x + 1, −y, −z + 1; (iii) x − 1, y, z. The molecules shown were taken into account for the evaluation of the intermolecular magnetic interactions by density functional theory (DFT) methods.

Regarding crystal packing, the centrosymmetric dimeric units are linked in a two-dimensional architecture (parallel to the *a-b* plane) through moderate O1–H1B^…^O8^iii^ (D^…^A: 2.954(3) Å; D–H^…^A: 152(3)°; iii = x − 1, y, z) and weak C4 –H4^…^O8^ii^ (D^…^A: 3.096(4) Å; D–H^…^A: 145.9°; ii = −x + 1, −y, −z + 1) hydrogen bonds (Figure 2; Table S2).

Further, intermolecular π···π stacking interactions are observed between intercalated aromatic rings of the *bipy* ligands, building layers that are parallel to the crystallographic *a-b* plane with distances between centroids of 3.7–3.8 Å (Figure 2), and between overlapping atoms of 3.426 and 3.430 Å for C3^…^C9^iv^ (iv = −x, −y, 1 − z) and C4^…^C6^ii^. Additionally, intramolecular hydrogen bonds, O1 –H1A^…^O7, are observed between the non-coordinated benzoate oxygen atom and a hydrogen atom from the coordinated water molecule (D^…^A: 2.723(3) Å; D–H···A: 153(3)°).

The intermolecular interactions are crucial for the crystal packing and organisation observed of the molecules in the solid state. The Cu1···Cu1^i^ (i = 1 − x, 1 − y, 1 − z) distance within the dimer is 5.602 Å, whereas the shortest intramolecular Cu···Cu distance is 5.888 Å for Cu1···Cu1^ii^, and occurs along the *a* axis. The intramolecular Cu···Cu contact is significantly longer than corresponding distances in related dimeric oxalate-bridged copper(II) species with *bipy* ligands, which present values in the range of 5.13–5.17° [11,14,45,48,57]. Finally, the copper(II) centres are displaced 0.01 Å from the principal mean plane formed by the O1, N1, O8, C18 and O5 atoms and their symmetry equivalents.

### 2.2. Thermal Analysis

In order to investigate the thermal stability of **1**, the complex was submitted to thermogravimetric (TG) and differential thermogravimetric (DTG) analyses with a heating rate of 10 °C min^−1^ under N_2_/O_2_ flow. The TG/DTG curves and the attribution of each decomposition step are shown in Figure S2 and Table S3, respectively. The thermal profile obtained for **1** shows three significant decomposition steps: in the first step, a mass loss of 5.6% refers to both coordinated water molecules (calc. 4.5%) in the range of 55–136 °C, followed by a second loss with a shoulder at 210 °C that suggest full decomposition of the complex. However, from the found and calculated mass contents it was not possible to distinguish which ligand was lost in these two final steps, although one would expect *bzt^−^* to leave the complex first when compared to *bipy* and *ox^2^^−^* due to the chelate effect [58,59]. The found and calculated values for the loss of *bzt^−^* and *bipy* ligands in step II, 62.5% and 68.9%, respectively, may indicate that their decomposition process continues to decompose in step III, which, in turn, starts at 305 °C. The weight loss observed for *ox^2^^−^* (15.3%), considered here the last ligand lost in step III, is above the calculated content of 10.9%, and this could correspond to *ox^2^^−^* being lost together with the remaining mass of *bzt^−^* and/or *bipy*.

Considering the final mass content at 900 °C, which corresponds to experimental and calculated total mass losses of 84.3% and 83.4%, respectively, one infers that the complex was decomposed generating CuO as the final product (experimental 16.4%; calculated 15.8%). This obtained oxide was confirmed by powder XRD of a sample of **1** treated at 900 °C, as shown in Figure S3.

### 2.3. Vibrational Spectroscopy

A comparative IR study of **1** and its starting materials is presented in Figure S4 and Table S4. The spectrum of **1** indicates deprotonation of both the benzoic and oxalic acids according to the quenching of the vibrational modes at 1424 cm^−1^, δ(COH)_COOH_, and 936 cm^−1^, β(OH)_COOH_, for H*bzt*, and also at 1264 cm^−1^ for δ(OH)_COOH_ in H*_2_ox*. The binding of the *O*-donor ligands to the copper(II) centre is additionally evidenced by the shift of the absorption bands to lower frequency, from 1685–1689 to 1648 cm^−1^, attributed to ν_as_(CO) for H*_2_ox* and H*bzt*, respectively. The arisal of an absorption band at 1379 cm^−1^, assigned to ν_as_(CO)_COO_−, also indicates the coordination of the *bzt^−^* and *ox^2^^−^* ligands [58,60,61,62]. The intense and broadened bands assigned as ν(OH) for the coordinated water molecules confirm the occurrence of hydrogen bonds (O1-H1B···O8^ii^ and O1-H1A···O7, Figure 2 and Table S2) in **1** [61].

The Raman spectrum recorded for **1** (Figure S5 and Table S5) is in accordance with the infrared analysis as far as the incorporation of the three ligands is concerned: bands at 366-619 cm^−1^, assigned to ν_s_(Cu-O) and ν_s_(Cu-N), were observed and indicate the coordination of *bipy*, *ox^2^^−^* and *bzt^−^* [63].

### 2.4. Electron Paramagnetic Resonance Spectroscopy (EPR)

**Figure 3:**
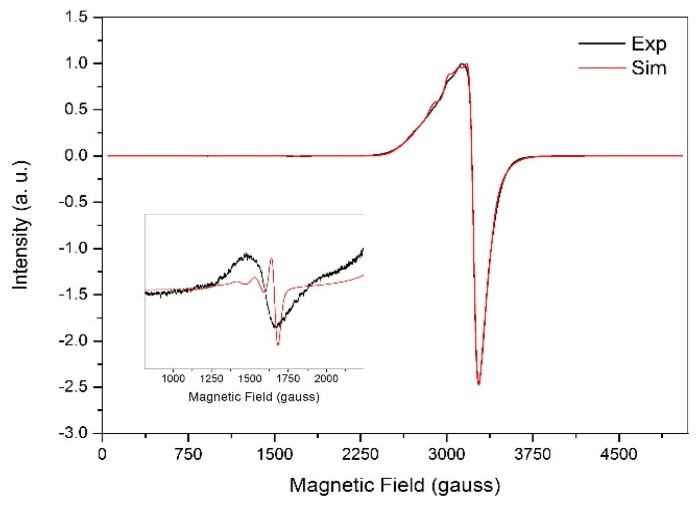
X-band EPR spectra for solid **1** (ground crystals) at 77 K with amplification (inset) for the half-field transition. Experimental spectrum in black and simulated spectrum in red.

The X-band EPR spectrum recorded for polycrystalline **1** (ground crystals) at 77 K, along with simulation (parameters in Table 2), gives an axial profile with *g*_⊥_ = 2.09 and *g*_||_ = 2.26, as presented in Figure 3. These values are expected for tetragonally-distorted copper(II) complexes with elongation along the *z*-axis, which is in good agreement with the longest bonds in the complex [Cu(1)–O(1), 2.425(3) Å; Cu(1)–O(8), 2.378(2) Å]. The computed *g*-values at DFT level (*g*_⊥_ = 2.06 and *g*_||_ = 2.21) are in excellent agreement with the experiment. The *g*_⊥_ < *g*_||_ relationship indicates $d_{x^{2}-y^{2}}$ as the singly occupied molecular orbital (SOMO), as suggested by the crystal structure [64,65]. The broadness of the EPR signal centred at 3230 G, which is due to the allowed transitions with *ΔM_s_* = ±1, agrees with the presence of magnetic interaction between the copper(II) centres in the dimer. The very weak half-field signal for the *ΔM_s_* = ±2 transitions, characteristic of copper(II) dimeric compounds with exchange interaction between the metal centres, is also observed as shown in the inset of Figure 3. Such transitions cannot be observed in *S* = ^1^/_2_ spin systems, being an irrefutable proof of the dimer formation in this particular case. The simulated axial zero field splitting (ZFS) parameter, *D*, translates to a Cu···Cu distance of 6.35 Å, which, considering the uncertainty introduced by the non-resolved hyperfine couplings, correlates well to the crystallographic Cu···Cu distance of 5.60 Å.

**Figure 4:**
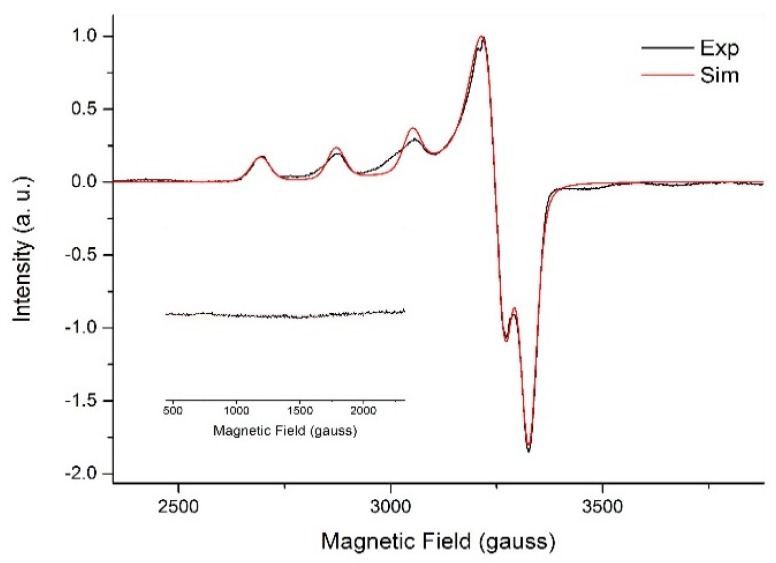
X-band EPR spectra recorded for **1** in frozen H_2_O/glycerol 9:1 (3.0 mmol L^−1^) solution at 77 K with amplification (inset) evidencing the lack of a half-field transition. Experimental spectrum in black and simulated spectrum in red.

The frozen solution X-band EPR spectrum of **1** was recorded at 77 K in H_2_O/glycerol (9:1). It is also axial with *g_x_*, *g_y_* (2.05 and 2.08) < *g*_z_ (2.26). These values are again in agreement with those reported for axial copper(II) complexes containing coordinated *ox*^2^*^−^* or water [66,67]. The four-line hyperfine structure is observed in Figure 4, and arises from the interaction between the unpaired electron of copper(II) (*S* = ^1^/_2_) and its nuclear spin (*I* = ^3^/_2_) [68].

The lack of a half-field transition, according to the inset in Figure 4, indicates that the dimeric structure of **1** is not maintained in solution. This proposition is corroborated by the simulated spectrum (red line in Figure 4, simulation parameter in Table 2), which was generated for a corresponding, hypothetical mononuclear complex in solution.

The proposition of possible structures for the mononuclear complexes formed in solution is difficult given the presence of four different ligands in equal proportion—this raises the possibility of a variety of different structures. Despite this possible structural variety, it is interesting to note that slow evaporation of the EPR solution of complex **1** (3.0 mmol L^−1^) leads to its regeneration as verified by the unit cell analysis of the resulting crystals.

### 2.5. UV/Vis Spectroscopy

The electronic spectra recorded for **1** in aqueous solutions of different concentrations, Figure S6, show a broad asymmetric band at 650–750 nm that can be attributed to *d-d* transitions in *d*^1^ centres. The assignment of the energy states involved in these transitions is difficult due to the lack of information about the structures and, therefore, the symmetries of the mononuclear complexes formed in solution, as suggested by EPR spectroscopy. Besides the *d-d* transitions, the electronic spectra show intense, higher energy bands below 310 nm which may be assigned to ligand-to-metal charge transfer and/or intra-ligand transitions [17,58,69].

### 2.6. Magnetic Properties and ab Initio Calculations

**Figure 5:**
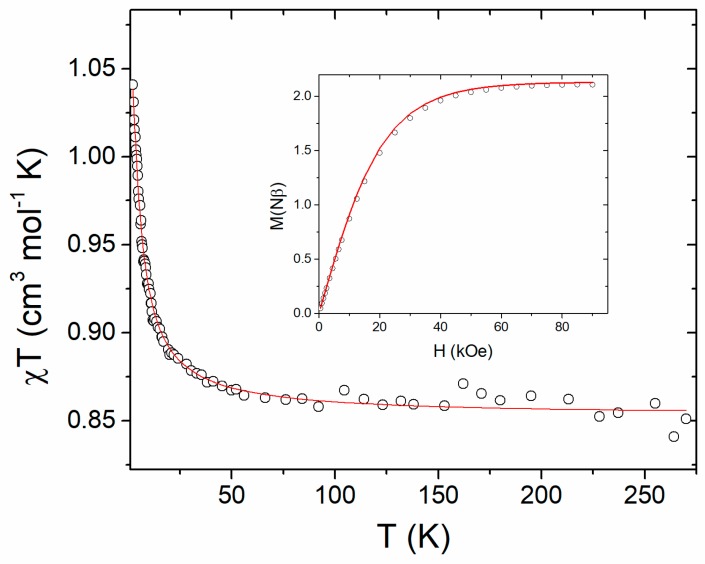
χ_M_T vs. T and M vs. H at 2.0 K (inset) for **1**. The solid lines correspond to the calculated curves using the best fit parameters described in the text.

The χ_M_T vs. T curve of **1**, as well as its field-dependent magnetisation at 2.0 K from a polycrystalline sample are shown in Figure 5.

The χ_M_T *versus* T plot shows that, in the high temperature range (50 < T < 280 K), χ_M_T is nearly constant (*ca.* 0.87 cm^3^ K mol^−1^), which is in good agreement with the calculated value (0.85 cm^3^ K mol^−1^) considering two copper(II) ions with *g* = 2.13, as obtained by simulation of the EPR spectra and by ab initio calculations. Upon cooling, there is a gradual increase of χ_M_T, reaching a maximum value of 1.04 cm^3^ K mol^−1^ at 2.8 K, which gives support to the existence of a ferromagnetic interaction.

Due to the dimeric nature of **1**, the magnetic susceptibility data were analysed considering an intramolecular magnetic interaction using the Hamiltonian H = −*J**S*****_1_.*S*_2_**, with ***S*_1_** = ***S*_2_** = 1/2. The magnetic susceptibility data were fitted using the DAVE software [70]. The best-fit parameters used were the average *g*-value = 2.14 and the magnetic exchange interaction (*J* = 2.3 cm^−1^), confirming that the magnetic exchange interaction between the two magnetic copper(II) ions through the oxalate bridge is weak and ferromagnetic.

The intermolecular through-bond and through-space magnetic interactions were initially neglected. However, since the intermolecular interactions are mediated by hydrogen bonds between water molecules and the oxalate bridge, and by π ·· π interactions between bipyridines, we have verified that the eventual through-bond magnetic interactions are either absent or of lower orders of magnitudes. Therefore, we chose the two pairs of molecules where the closest Cu^II^ ions lie at 5.88 and 7.22 Å, respectively (see Figure 2, Section 2.1 and Section 3.4). The computed magnetic-coupling constants for the two pairs are antiferromagnetic, and their values are 0.092 and 0.085 cm^−1^ for the first and second pair, respectively. The strength of these intermolecular interactions is one order of magnitude lower than the intramolecular one. However, such a value cannot be neglected and, therefore, it was included in the fit. In order to prevent over-parametrisation, the intermolecular magnetic interaction was obtained considering the mean field approximation and was fixed to the value obtained from DFT calculation while the magnetic exchange interaction and average *g*-value were allowed to vary. The best fit parameters obtained were *J* = 2.9 cm^−1^, average *g*-value = 2.13 and intermolecular magnetic interaction *λ* = −0.09 cm^−1^ (fixed) (Figure 5). The agreement factor *R*, defined as *R* = Σ[(χ_M_T)_obs_ − (χ_M_T)_cal_]^2^ / Σ[(χ_M_T)^2^_obs_, is equal to 1.8 × 10^−5^, and is affected by the noisier data for temperatures above 60 K; otherwise, at lower temperatures, the fitting is very good. The inset of Figure 5 shows the field-dependent magnetisation at 2.0 K and the simulated curve using the same Hamiltonian and parameters as the fit of χT vs. T, confirming the intramolecular ferromagnetic interaction. The dipolar through-space magnetic interactions have also been evaluated for the Cu^II^ ion considering all the magnetic centres in the crystal packing, which are less than 20 Å away from the centre. The interactions were computed using the method described by Bencini and Gatteschi, employing the directions and values of the computed ab initio *g*-tensor [71]. The isotropic part of such computed magnetic exchange is 0.00019 cm^−1^, four orders of magnitude lower than the intramolecular one, and three orders of magnitude lower than the through-bond intermolecular one. As a consequence, unlike the through-bond exchange, the dipolar interaction was not included in the fits.

**Figure 6:**
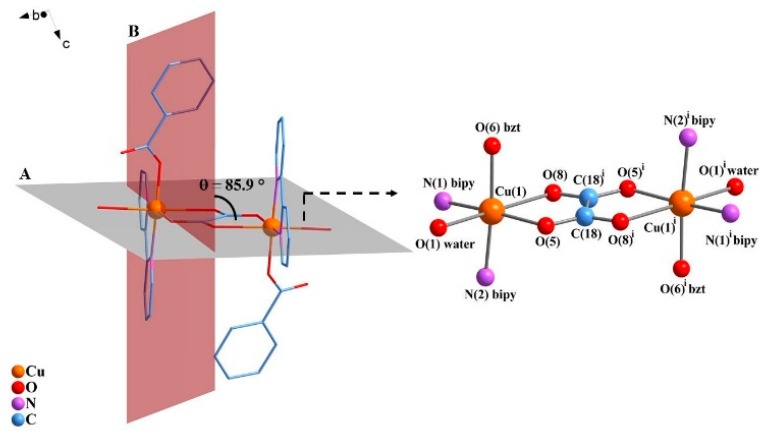
Representation of the dihedral angle (*θ*) between plane A, which contains the *ox*^2−^ bridge, and plane B, which contains the $d_{x^{2}-y^{2}}$ orbitals. Hydrogen atoms were omitted for clarity. Symmetry code (i): 1 − x, 1 − y, 1 − z.

Symmetry considerations applied to the magnetic orbitals of the copper(II) centres help to understand the nature of the magnetic interactions in **1.** Structural parameters, such as the Cu···Cu distance (*d_Cu_···d_Cu_*) across the bridging oxalate, the distance between the copper(II) ions and the plane described by oxygen and carbon atoms of the oxalate group, and the dihedral angle (*θ*) between the plane of the containing the bridging oxalate and the equatorial plane of the copper(II) complex, significantly influence the nature and the magnitude of the magnetic interactions. All these data can be useful in predicting the nature of the magnetic exchange [8,9,10,72]. For oxalate-bridged copper(II) compounds it is well known, both from experiments and theoretical calculations, that if the symmetry-related $d_{x^{2}-y^{2}}$ magnetic orbitals are orthogonal to the oxalate-bridge, the compound can exhibit a weak ferromagnetic interaction. In **1**, the plane of the bridging oxalate ion is formed by O(5), O5^i^, O8, O8^i^, C18 and C18^i^ (plane **A** in Figure 6). So, according to the magnetic data, the magnetic $d_{x^{2}-y^{2}}$ orbitals were expected to be located in the plane formed by O5 from *ox^2^^−^*, O6 from *bzt^−^* and N1 and N2 from *bipy* (plane **B** in Figure 6). In this case, the magnetic orbitals for both copper(II) centres would be parallel to each other and almost orthogonal to the oxalate bridge, the so-called ‘accidental orthogonality’, with a dihedral angle of approximately 86°, as reported for other ferromagnetic oxalate-bridged copper(II) dinuclear complexes (Table 3) [8].

There is also a reported correlation between the Cu–O_axial_–C_ox_ bond angle (*α*) and the nature of the magnetic interaction for oxalate-bridged copper(II) complexes that present weak magnetic interaction. For *α* < 109.5°, it is highly probable that the magnetic interaction will be ferromagnetic. On the other hand, if *α* > 109.5°, the magnetic interaction will most likely be antiferromagnetic [8,9]. For **1**, the asymmetrical coordination of the oxalate ligand is assured by the bond lengths Cu1–O5 and Cu1–O8_axial_ of 1.973(18) and 2.378(2) Å, respectively, and the value of the *α* angle, given by Cu1–O8_axial_–C18^i^, is equal to 107.7 ° Based on this discussion, the comparison of the values of *d_Cu_···d_Cu_*, *θ* and *α* for complexes similar to **1** that exhibit weak ferro- or antiferromagnetic exchange interactions, as determined by temperature-dependent magnetic susceptibility measurements, are shown in Table 3. These structural parameters were obtained from structures reported in the literature as Crystallographic Information Files (CIF files) using Mercury software [73]. The weak magnetic interaction determined for **1** is in the same range reported for other binuclear complexes with this type of structural arrangement.

**Figure 7:**
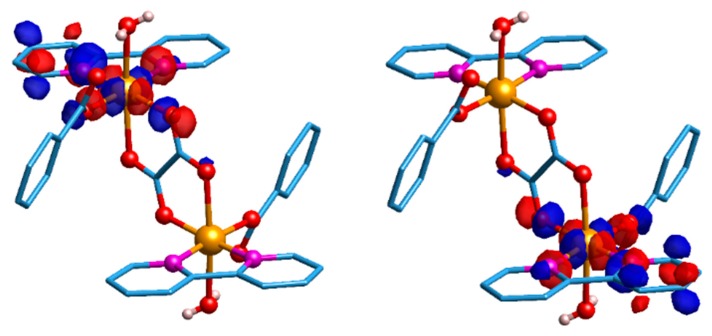
Graphic representation of the two empty broken symmetry (BS) magnetic orbitals for complex **1** (left panel, spin-up; right panel, spin-down). The electron density iso value was set to 0.04 e/(a_0_)^3^. Light-blue: carbon; white: hydrogen; red: oxygen; orange: copper; pink: nitrogen.

To give support to these magnetic orbital symmetry considerations, DFT calculations were performed using ORCA 4.0.1 software [74]. The magnetic orbitals are sharply localised and the local $d_{x^{2}-y^{2}}$ character is confirmed for each copper ion (see Figure 7). Indeed, they are parallel to each other, but perpendicular to the oxalate plane for both broken symmetry (BS) magnetic orbitals. Smaller values of electron density cut-off (≤0.05 e^−^/a_0_^3^) evidenced a small amount of delocalisation on the second magnetic centre. However, such a delocalised density corresponds to a $d_{xz}$ orbital, which, being orthogonal to the local $d_{x^{2}-y^{2}}$ orbital, may lead to a ferromagnetic pathway (see Figure S7) [75]. The calculations have also shown that the spin density computed on each of the copper(II) centres is essentially the same, as expected from molecular symmetry considerations. The theoretically predicted *J* value of +7.72 cm^−1^ is of the correct sign and order of magnitude, and therefore is in good agreement with the experimental *J* value, +2.9 cm^−1^, confirming that the ferromagnetic interaction is weak.

## 3. Materials and Methods

### 3.1. Materials and Physical Measurements

All chemicals and solvents were used as acquired, without further purification. Chemicals were purchased from Sigma–Aldrich (St. Luis, MO, USA) and Merck (Merck, Darmstadt, Germany) (purity grade: 99.5%) and solvents from Sigma–Aldrich (St. Luis, MO, USA) (purity grade: 98.8%–99.0%). Elemental (C, H, N) analyses were performed on a Thermo Fisher Scientific Flash EA CHNS-O 1112 series element analyser (Thermo Fischer Scientific Inc., Waltham, MA, USA). Copper dosage was performed on an atomic absorption spectrophotometer (GBC Scientific Equipment Pty Ltd, Hampshire, IL, USA), GBC Avanta, equipped with a flame atomiser. The crystalline phase purity of the sample was examined by powder X-ray diffraction (PXRD) using a Shimadzu XRD-6000 diffractometer (Shimadzu Industrial Systems Co., Ltd., Tokyo, Japan) equipped with Cu-Kα radiation (*λ* = 1.5418 Å) in a 2θ range of 10–60°. The voltage and current applied were 40 kV and 30 mA. The simulated diffractogram was performed using Mercury software [73] from the data in the CIF file of **1**. Infrared (IR) spectra were recorded with a BIORAD FTS 3500GX spectrophotometer (Bio-Rad Laboratory, Hercules, CA, USA) from KBr pellets in the range of 400–4000 cm^−1^, with a resolution of 4 cm^−1^ in 32 scans. The Raman spectrum was recorded with a Renishaw Raman image spectrophotometer (Renishaw Plc., New Mills, United Kingdom) coupled with a Leica optical microscope in the range of 100–4000 cm^−1^; a He-Ne laser (632.8 nm) was employed. The analysed area of the sample was of 1 μm^2^ with an applied voltage of 0.2 mW. Electron paramagnetic resonance (EPR) measurements were carried out in the solid-state (powdered crystals) and in solution (3.0 mmol L^−1^ in H_2_O/glycerol 9:1) using an X-band Bruker ELEXSYS MX-micro spectrometer (Bruker BioSpin Corporation, Billerica, MA, USA) operating at 9.5 GHz. Simulations were carried out with the EasySpin software package [76]. Magnetic measurements were performed on a PPMS Quantum Design magnetometer (Quantum Design North America, San Diego, CA, USA) in the temperature range of 2–300 K—the powdered sample was pressed into a pellet. Temperature dependence of magnetisation was measured with an applied external magnetic field of 1 kOe between 2–60 K and 10 kOe above 60 K. Diamagnetic corrections were made using Pascal constants [77]. Electronic spectra were recorded on a UV/Vis Varian Cary 50 spectrophotometer (Agilent Technologies Inc., Santa Clara, CA, USA) in the range of 190–800 nm. Thermogravimetric (TG) and differential thermogravimetric (DTG) analyses were carried out on a NETZSCHSTA 449 F3 Jupiter analyser (Netzsch, Selb, Germany); the experiment was conducted under O_2_/N_2_ (flow rate of 50 mL min^−1^) in the range of 20–900 °C at a heating rate of 10 °C min^−1^.

### 3.2. Synthesis of Compound **1**

A solution of benzoic acid (0.122 g, 1.00 mmol) and diethylamine (105 μL, 1.00 mmol) in 10 mL of methanol was added to a suspension of copper(II) acetate monohydrate (0.199 g, 1.00 mmol) in 40 mL of methanol at 70 °C. The reaction mixture was stirred for 10 min leading to a bluish-green solution, which then received the addition of a colourless mixture of oxalic acid dihydrate (0.063 g, 0.50 mmol) and diethylamine (105 μL, 1.00 mmol) in 10 mL of methanol. A light-blue solution was immediately formed and was stirred for 10 min. Finally, the addition of a light-yellow solution of 2,2′-bipyridine (0.156 g, 1.00 mmol) in 10 mL of methanol, followed by stirring for 30 min, led to a change of colour to dark-blue. Bluish-green prismatic crystals were obtained after solvent evaporation at room temperature for 36 days (0.3640 g, 90% yield based on copper(II) acetate monohydrate). Solubility: slightly soluble in water, methanol, and glycerol. Elemental analysis calculated (%) for C_36_H_30_Cu_2_N_4_O_10_: C 53.66, H 3.75, N 6.95, Cu 15.77; found: C 53.26, H 3.77, N 7.14, Cu 16.39. IR (KBr, cm^−1^, s = strong, m = medium, w = weak): ν(O-H) = 3460(m); ν(C-O, COO^−^) = 1648, 1379(*s*); ν(C=C and C=N) = 1602–1445(m, w); δ(C-H) = 1305–1019(w); β(C-H) = 779–675(m). Raman (cm^−1^): ν = 3054(m) (O-H); ν(CO)_COO_− = 1597 (s); ν(C=C and C=N) = 1597–1494(s, m); δ(CCH) = 1268(w); δ(OCO) = 843(w); ν(Cu=O and Cu=N) = 619–366(w).

### 3.3. X-ray Crystallographic Data Collection and Refinement

A bluish-green single crystal of **1** was mounted on a micromount-type mesh (Mitegen). The data was obtained on a Bruker D8 Venture diffractometer equipped with a Photon 100 CMOS detector, Mo-Kα radiation (*λ* = 0.71073 Å) and graphite monochromator. Diffraction data were measured at 302(2) K and processed using the APEX3 program [78]. The structure was determined by the intrinsic phasing routines in the SHELXT program [79] and refined by full-matrix least-squares methods, on F^2^’s using SHELXL program [80,81] in the WinGX suite [82]. The non-hydrogen atoms were refined with anisotropic thermal parameters. All hydrogen atoms were located in difference Fourier maps and were refined isotropically and freely. Molecular diagrams were prepared using ORTEP3 [82] and Mercury software [73]. The crystal data collection and refinement parameters are summarised in Table 4.

### 3.4. Theoretical Methods

Density functional calculations were performed with the quantum chemistry software ORCA 4.0.1 [74]. The X-ray structure of **1** was employed without any further geometry optimisation. The PBE0 [83] hybrid exchange-correlation functional was chosen. The basis sets were: def2-TZVPP [84] on Cu, N, O and C, and def2-SVP on H. The broken symmetry (BS) approach [85] was used to extract the magnetic-coupling constants. Single point calculations on both the triplet and the broken symmetry state were performed. Hence, within the Heisenberg Hamiltonian H = −*JS*_1_·*S*_2_, the value of the exchange constant was computed within the following formula: *J* = −(*E*_HS_ − *E*_BS_)/(2*S*_1_*S*_2_), where HS is the triplet (*S* = 1). Within the same approach, the intermolecular exchange interaction was also computed in two dimer models. Both models were made using two molecular units of **1**, as extracted from the crystal structure—the two molecular pairs whose closest Cu ions lay at 5.88 Å and 7.22 Å away from each other, respectively. The two pairs were chosen due to their Cu···Cu distance below 10 Å. Moreover, in both molecules the first pair interacts via hydrogen bonds between the oxalate bridge and the water molecules, while the second pair interacts through π-π stacking of the bipyridine ligands (see Section 3.1), which could promote a non-negligible superexchange path via π orbital overlap. All the other Cu atoms in the crystal are at a larger distance and, as a consequence, they were neglected in a first approximation. The exchange constant was computed by converging on the quintuplet and on the singlet states, i.e., the two states that arise from the two ground triplets ferromagnetically and antiferromagnetically coupled. The g-matrix of a single copper(II) atom was also computed by DFT methods. For this purpose, one of the two copper(II) atoms of the dimer was substituted in silico for its diamagnetic equivalent zinc(II). The *g*-matrix was obtained within the coupled-perturbed self-consistent field (CPSCF) approach [86] as implanted in Orca.

## 4. Conclusions

A new dinuclear copper(II) complex was synthesised and characterised. The crystal structure showed that [[{Cu(*bipy*)(*bzt*)(OH_2_)}_2_(μ-*ox*)] (**1**) is centrosymmetric and contains two rather bulky ligands, benzoate and 2,2′-bipyridine, while the majority of the complexes of this class contain just one bulky ligand. The magnetic interaction predicted by EPR was confirmed by magnetic measurements and indicated ferromagnetic coupling between the metal centres. The determined *J* value was supported by DFT calculations. Therefore, the present study highlights the importance of the oxalate bridge as a magnetic-exchange pathway, and how bulky ancillary ligands can affect the magnetic response.

## Figures and Tables

**Table 1:** Selected bond lengths (Å) and angles (°) for **1**, with estimated standard deviations in parentheses.

**Bond Length (Å)**			
Cu(1)–O(1)	2.426(3)	Cu(1)–N(2)	2.006(2)
Cu(1)–O(5)	1.9732(19)	O(6)–C(11)	1.263(3)
Cu(1)–O(8)	2.378(2)	O(7)–C(11)	1.230(3)
Cu(1)–O(6)	1.9425(19)	C(18)–O(5)	1.260(3)
Cu(1)–N(1)	2.008(2)	C(18) ^i^–O(8)	1.230(3)
**Bond Angle (°)**			
O(5)–Cu(1)–O(8)	76.62(7)	O(5)–Cu(1)–N(2)	93.67(8)
O(6)–Cu(1)–O(1)	89.46(9)	N(1)–Cu(1)–O(8)	95.24(7)
O(1)–Cu(1)–O(5)	94.26(8)	N(2)–Cu(1)–O(8)	94.08(8)
O(1)–Cu(1)–O(8)	170.00(7)	N(1)–Cu(1)–N(2)	80.56(9)
O(6)–Cu(1)–O(5)	92.77(9)	N(1)–Cu(1)–O(1)	94.26(8)
O(6)–Cu(1)–O(8)	86.93(9)	N(2)–Cu(1)–O(1)	90.55(9)
O(6)–Cu(1)–N(1)	93.00(9)	H(1B)–O(1)–H(1A)	104(3)
O(6)–Cu(1)–N(2)	173.55(9)	O(8) ^i^–C(18)–O(5)	124.4(2)
O(5)–Cu(1)–N(1)	169.75(9)	O(7)–C(11)–O(6)	125.7(3)

Symmetry code (i): 1 − x, 1 − y, 1 − z.

**Table 2:** EPR spectrum simulation parameters for the frozen solution and solid powder spectra at 77 K.

Sample	*g*-Matrix			*A*-Matrix/MHz			*D*-Matrix/MHz	
	*g_x_*	*g_y_*	*g_z_*	*A_x_*	*A_y_*	*A_z_*	*D*	*E*
Solid	2.079	2.079	2.278	<100	<100	>360	350	65.5
Frozen solution	2.053	2.078	2.262	<100	<100	555.1	-	-

**Table 3:** Selected magnetostructural parameters for oxalate-bridged copper(II) complexes.

Compound ^(a)^	*d_Cu__⋅⋅⋅Cu_* (Å) ^(b)^	*θ* (°) ^(c)^	*α* (°) ^(d)^	*J* (cm^−1^) ^(e)^	Reference
[{Cu(*bipy*)(*bzt*)(OH_2_)}_2_(μ-*ox*)]	5.60	85.9	107.7	+2.3	Complex **1**
[{Cu(dpyam)_2_}_2_(μ-ox)](BF_4_)_2_·3H_2_O	5.74	86.1	-	+3.38	[44]
[{Cu(dpyam)_2_}_2_(μ-ox)](ClO_4_)_2_·3H_2_O	5.75	77.0	-	+2.42	[44]
[{Cu(prbipy)}_2_(μ-ox)]·4H_2_O	5.46	78.7	107.4	+3.22	[9]
[{Cu(bpca)(H_2_O)}_2_(μ-ox)]·2H_2_O	5.63	80.7	106.8	+1.1	[7]
[{Cu(bpca)}_2_(μ-ox)]	5.44	86.3	107.4	+1.0	[7,49]
[{Cu(bpcam)(H_2_O)}_2_(μ-ox)]	5.68	81.6	106.6	+0.75	[8]
[{Cu(dien)}_2_(μ-ox)](NO_3_)_2_	5.14	4.6	110.2	−6.5	[45]
[{Cu(3-ampy)}_2_(μ-ox)]_n_	5.46	4.8	111.0	−1.3	[10]
[{Cu(4-ampy)}_2_(μ-ox)]_n_	5.66	2.4	109.7	−1.1	[10]

^(a)^ Ligand abbreviations: *ox* = oxalate, *bipy* = 2,2′-bipyridine; *bzt* = benzoate; prbipy = *N*-(2-pyrazinyl)-4,4′-bipyridinium; bpca = bis(2-pyridylcarbonyl)amidate; bpcam = bis(2-pyrimidyl)amidate; dpyam = (di-2-pyridylamine); dien = diethylenediamine; ampy = aminopyridine. ^(b)^ Distance between copper(II) centres mediated by the oxalate bridge. ^(c)^ Dihedral angle between the oxalate plane and the plane containing the magnetic orbital of the complex. ^(d)^ Cu−O_axial_−C_ox_ bond angle measured only when the oxalate is asymmetrically coordinated. ^(e)^ Magnetic coupling parameter.

**Table 4:** Crystal data and refinement parameters for compound **1**.

Formula	C_36_H_30_Cu_2_N_4_O_10_
Formula weight	805.72 g mol^−^^1^
Temperature (K)	302(2)
Wavelength (Å)	0.71073 Å
Crystal system	Monoclinic
Space group	*P*2_1_/*n* (equiv. to no 14)
Z/calculated density	2/1.593 Mg m^−3^
*a* (Å)	7.1994(3)
*b* (Å)	10.0894(4)
*c* (Å)	23.1941(10)
*β* (°)	94.385(2)
Unit cell volume (Å^3^)	1679.83(12)
Absorption coefficient *μ* (mm^−1^)	1.333
F(000)	824
Crystal size (mm)/colour	0.175 × 0.087 × 0.067/bluish-green
*θ* range (°)	2.9–25.0
*h, k, l* ranges	±8, ±11, ±27
Completeness to *θ* = 25°	99.9%
Total reflections/unique reflections/R_int_	51619/2953/0.069
No of parameters/restraints	243/2
Goodness-of-fit on F^2^ (GOF)	1.037
R_1_, *w*R_2_ (all data) ^b^	0.052, 0.085
R_1_, *w*R_2_ [*I* > 2σ(*I*)] ^a,b^	0.033, 0.078
Δρ*_maximum_/*Δρ*_minimum_*(eÅ^−3^)	0.29/−0.17

^a^ R_1_ = Σ||Fo| − |Fc||(Σ|Fo|)^−1^; ^b^ *w*R_2_ = {Σ[w(Fo^2^ − Fc^2^)^2^[Σ(Fo^2^)^2^]^−1^}^1/2^.

## Supplementary Materials

The following material is available online, Figure S1: Comparison between simulated and experimental PXRD patterns of complex **1** (scan velocity: 0.005° s^−1^), Figure S2: Thermogravimetric analysis (TG and DTG) profiles obtained for **1** in O_2_/N_2_ as carrier gas and temperature range of 20 to 900 °C, Figure S3: Comparison between simulated and experimental PXRD patterns of CuO (scan velocity: 0.005° s^−1^), Figure S4: Infrared spectra of **1** and its starting materials, H*_2_ox·2*H*_2_O*, H*bzt* and *bipy* recorded from KBr pellets, Figure S5: Raman spectrum recorded for **1** with exposure time of 10 s and power at 50 % (laser: He-Ne, 632.8 nm), Figure S6: UV-Vis absorption spectra recorded for complex **1** at 3.00, 2.50, 2.00, 1.50 and 0.75 mmol L^−1^ in water, Figure S7: Graphic representation of the two occupied broken symmetry (BS), spin-up and spin-down, magnetic orbitals for complex **1.** The electron density iso value was set to 0.04 e/(a_0_)^3^. Light-blue: carbon; white: hydrogen; red: oxygen; orange: copper; pink: nitrogen, Table S1: Values of 2θ angles for the experimental and simulated PXRD diffractograms of **1** from 10 to 30° displaying a difference of ca 0.1°, Table S2: Hydrogen bond parameters (Å, °), Table S3: Thermal data for **1**, Table S4: Tentative assignments for the IR (cm^−1^) spectrum recorded for complex **1**, Table S5: Tentative assignments for the Raman scattering (cm^−1^) spectrum recorded for complex **1**. CCDC 1971374 contains the supplementary crystallographic data in CIF format, and this data can be obtained free of charge at http://www.ccdc.cam.ac.uk/structures.

## References

[1] Huang K.-B., Chen Z.-F., Liu Y.-C., Xie X.-L., Liang H. (2015). Dihydroisoquinoline copper(II) complexes: Crystal structures, cytotoxicity, and action mechanism. Rsc. Adv..

[2] Usman M., Arjmand F., Khan R.A., Alsalme A., Ahmad M., Bishwas M.S., Tabassum S. (2018). Tetranuclear cubane Cu4O4 complexes as prospective anticancer agents: Design, synthesis, structural elucidation, magnetism, computational and cytotoxicity studies. Inorg. Chim. Acta.

[3] Sanyal R., Kundu P., Rychagova E., Zhigulin G., Ketkov S., Ghosh B., Chattopadhyay S.K., Zangrando E., Das D. (2016). Catecholase activity of Mannich-based dinuclear CuII complexes with theoretical modeling: New insight into the solvent role in the catalytic cycle. New J. Chem..

[4] Parween A., Naskar S., Mota A.J., Espinosa Ferao A., Chattopadhyay S.K., Rivière E., Lewis W., Naskar S. (2017). C i -Symmetry, [2 × 2] grid, square copper complex with the *N*^4^,*N*^5^-bis(4-fluorophenyl)-1H-imidazole-4,5-dicarboxamide ligand: Structure, catecholase activity, magnetic properties and DFT calculations. New J. Chem..

[5] Castro I., Calatayud M.L., Yuste C., Castellano M., Ruiz-García R., Cano J., Faus J., Verdaguer M., Lloret F. (2019). Dinuclear copper(II) complexes as testing ground for molecular magnetism theory. Polyhedron.

[6] Dutta D., Nath H., Frontera A., Bhattacharyya M.K. (2019). A novel oxalato bridged supramolecular ternary complex of Cu(II) involving energetically significant π-hole interaction: Experimental and theoretical studies. Inorg. Chim. Acta.

[7] Calatayud M.A.L., Castro I., Sletten J., Lloret F., Julve M. (2000). Syntheses, crystal structures and magnetic properties of chromato-, sulfato-, and oxalato-bridged dinuclear copper(II) complexes. Inorg. Chim. Acta.

[8] Cangussu D., Stumpf H.O., Adams H., Thomas J.A., Lloret F., Julve M. (2005). Oxalate, squarate and croconate complexes with bis(2-pyrimidylcarbonyl)amidatecopper(II): Synthesis, crystal structures and magnetic properties. Inorg. Chim. Acta.

[9] Zheng A.-L., Ju Z.-F., Li W., Zhang J. (2006). Ferromagnetic dinuclear copper(II) complex based on bridging oxalate and bipyridinium ligands. Inorg. Chem. Commun..

[10] Castillo O., Luque A., Román P., Lloret F., Julve M. (2001). Syntheses, Crystal Structures, and Magnetic Properties of One-Dimensional Oxalato-Bridged Co(II), Ni(II), and Cu(II) Complexes with n-Aminopyridine (*n* = 2−4) as Terminal Ligand. Inorg. Chem..

[11] Julve M., Gleizes A., Chamoreau L.M., Ruiz E., Verdaguer M. (2018). Antiferromagnetic Interactions in Copper(II) µ-Oxalato Dinuclear Complexes: The Role of the Counterion. Eur. J. Inorg. Chem..

[12] Zheng L.-Y., Chi Y.-H., Liang Y., Cottrill E., Pan N., Shi J.-M. (2018). Green and mild oxidation: From acetate anion to oxalate anion. J. Coord. Chem..

[13] Carranza J., Brennan C., Sletten J., Vangdal B., Rillema P., Lloret F., Julve M. (2003). Syntheses, crystal structures and magnetic properties of new oxalato-, croconato- and squarato-containing copper(II) complexes. New J. Chem..

[14] Melnic E., Kravtsov V.C., Krämer K., van Leusen J., Decurtins S., Liu S.-X., Kögerler P., Baca S.G. (2018). Versatility of copper(II) coordination compounds with 2,3-bis(2-pyridyl)pyrazine mediated by temperature, solvents and anions choice. Solid State Sci..

[15] Golchoubian H., Samimi R. (2017). Solvato- and thermochromism study in oxalato-bridged dinuclear copper(II) complexes of bidentate diamine ligands. Polyhedron.

[16] Royappa A.T., Royappa A.D., Moral R.F., Rheingold A.L., Papoular R.J., Blum D.M., Duong T.Q., Stepherson J.R., Vu O.D., Chen B. (2016). Copper(I) oxalate complexes: Synthesis, structures and surprises. Polyhedron.

[17] Świtlicka-Olszewska A., Machura B., Mroziński J., Kalińska B., Kruszynski R., Penkala M. (2014). Effect of *N*-donor ancillary ligands on structural and magnetic properties of oxalate copper(ii) complexes. New J. Chem..

[18] Miyazato Y., Asato E., Ohba M., Wada T. (2016). Synthesis and Characterization of a Di-µ-oxalato Tetracopper(II) Complex with Tetranucleating Macrocyclic Ligand. B. Chem. Soc. Jpn.

[19] Yuste C., Cañadillas-Delgado L., Labrador A., Delgado F.S., Ruiz-Pérez C., Lloret F., Julve M. (2009). Low-Dimensional Copper(II) Complexes with the Trinucleating Ligand 2,4,6-Tris(di-2-pyridylamine)-1,3,5-triazine: Synthesis, Crystal Structures, and Magnetic Properties. Inorg. Chem..

[20] Vicente R., Escuer A., Solans X., Font-Bardía M. (1996). Synthesis, magnetic behaviour and structural characterization of the alternating hexanuclear copper(II) compound [Cu_6_(tmen)_6_(µ-N_3_)_2_(µ-C_2_O_4_)_3_(H_2_O)_2_][ClO_4_]_4_·2H_2_O (tmen = Me_2_NCH_2_CH_2_NMe_2_). J. Chem. Soc. Dalton Trans..

[21] Nakahata D.H., de Paiva R.E.F., Lustri W.R., Ribeiro C.M., Pavan F.R., da Silva G.G., Ruiz A.L.T.G., de Carvalho J.E., Corbi P.P. (2018). Sulfonamide-containing copper(II) metallonucleases: Correlations with in vitro antimycobacterial and antiproliferative activities. J. Inorg. Biochem..

[22] Maxim C., Ferlay S., Train C. (2011). Binuclear heterometallic M(III)–Mn(II) (M = Fe, Cr) oxalate-bridged complexes associated with a bisamidinium dication: A structural and magnetic study. New J. Chem..

[23] Glerup J., Goodson P.A., Hodgson D.J., Michelsen K. (1995). Magnetic Exchange through Oxalate Bridges: Synthesis and Characterization of (mu-Oxalato)dimetal(II) Complexes of Manganese, Iron, Cobalt, Nickel, Copper, and Zinc. Inorg. Chem..

[24] Baruah B., Golub V.O., O’Connor C.J., Chakravorty A. (2003). Synthesis of Oxalato-Bridged (Oxo)vanadium(IV) Dimers Using L-Ascorbic Acid as Oxalate Precursor: Structure and Magnetism of Two Systems. Eur. J. Inorg. Chem..

[25] Bratsos I., Serli B., Zangrando E., Katsaros N., Alessio E. (2007). Replacement of Chlorides with Dicarboxylate Ligands in Anticancer Active Ru(II)-DMSO Compounds:  A New Strategy That Might Lead to Improved Activity. Inorg. Chem..

[26] Adamo C., Barone V., Bencini A., Totti F., Ciofini I. (1999). On the Calculation and Modeling of Magnetic Exchange Interactions in Weakly Bonded Systems:  The Case of the Ferromagnetic Copper(II) μ_2_-Azido Bridged Complexes. Inorg. Chem..

[27] Calzado C.J., Cabrero J., Malrieu J.P., Caballol R. (2002). Analysis of the magnetic coupling in binuclear complexes. I. Physics of the coupling. J. Chem. Phys..

[28] Calzado C.J., Cabrero J., Malrieu J.P., Caballol R. (2002). Analysis of the magnetic coupling in binuclear complexes. II. Derivation of valence effective Hamiltonians from ab initio CI and DFT calculations. J. Chem. Phys..

[29] Xu Z., Thompson L.K., Miller D.O. (1997). Dicopper(II) Complexes Bridged by Single N−N Bonds. Magnetic Exchange Dependence on the Rotation Angle between the Magnetic Planes. Inorg. Chem..

[30] Thompson L.K., Tandon S.S., Lloret F., Cano J., Julve M. (1997). An Azide-Bridged Copper(II) Ferromagnetic Chain Compound Exhibiting Metamagnetic Behavior. Inorg. Chem..

[31] Groom C.R., Bruno I.J., Lightfoot M.P., Ward S.C. (2016). The Cambridge Structural Database. Acta Cryst..

[32] Jurić M., Pajić D., Žilić D., Rakvin B., Milić D., Planinić P. (2015). Synthesis, crystal structures and magnetic properties of the oxalate-bridged single Cu^II^Cu^II^ and cocrystallized Cu^II^Zn^II^ systems. Three species (CuCu, CuZn, ZnZn) in the crystalline lattice. Polyhedron.

[33] Goswami S., Singha S., Saha R., Singha Roy A., Islam M., Kumar S. (2019). A bi-nuclear Cu(II)-complex for selective epoxidation of alkenes: Crystal structure, thermal, photoluminescence and cyclic voltammetry. Inorg. Chim. Acta.

[34] Oliveira W.X.C., Pereira C.L.M., Pinheiro C.B., Lloret F., Julve M. (2018). Oxotris(oxalate)niobate(V): An oxalate delivery agent in the design of building blocks. J. Coord. Chem..

[35] Samimi R., Golchoubian H. (2017). Dinuclear copper(II) complexes with ethylenediamine derivative and bridging oxalato ligands: Solvatochromism and density functional theory studies. Transit. Met. Chem..

[36] Calatayud M.L., Orts-Arroyo M., Julve M., Lloret F., Marino N., De Munno G., Ruiz-García R., Castro I. (2018). Magneto-structural correlations in asymmetric oxalato-bridged dicopper(II) complexes with polymethyl-substituted pyrazole ligands. J. Coord. Chem..

[37] Bahemmat S., Neumüller B., Ghassemzadeh M. (2015). One-Pot Synthesis of an Oxalato-Bridged CuII Coordination Polymer Containing an in Situ Produced Pyrazole Moiety: A Precursor for the Preparation of CuO Nano structures. Eur. J. Inorg. Chem..

[38] Liu C., Abboud K.A. (2015). Crystal structures of [mu]-oxalato-bis[azido(histamine)copper(II)] and [mu]-oxalato-bis[(dicyanamido)(histamine)copper(II)]. Acta Cryst..

[39] Min K.S., Suh M.P. (2000). Self-Assembly, Structures, and Magnetic Properties of Ladder-Like Copper(II) Coordination Polymers. J. Solid State Chem..

[40] Hökelek T., Ünaleroğglu C., Mert Y. (2000). Crystal Structure of [Bis(*N*,*N*,*N*’,*N*’-tetramethylethylenediamine)-*O*,*O*’-μ-*O*,*O*’-oxalato]dihydroxy Dicopper(II). Anal. Sci..

[41] Huang W., Ogawa T. (2009). Spectral and structural studies of a new oxalato-bridged dinuclear copper(II) complex having two 3-(thiophen-2-yl)-1,10-phenanthroline ligands in a trans configuration. Inorg. Chim. Acta.

[42] Thuéry P. (2014). Increasing Complexity in the Uranyl Ion–Kemp’s Triacid System: From One- and Two-Dimensional Polymers to Uranyl–Copper(II) Dodeca- and Hexadecanuclear Species. Cryst. Growth Des..

[43] Du M., Guo Y.-M., Chen S.-T., Bu X.-H., Ribas J. (2003). Crystal structures, spectra and magnetic properties of di-2-pyridylamine (dpa) Cu^II^ complexes [Cu(dpa)_2_(N_3_)_2_]·(H_2_O)_2_ and [Cu_2_(μ-ox)(dpa)_2_(CH_3_CN)_2_](ClO_4_)_2_. Inorg. Chim. Acta.

[44] Youngme S., van Albada G.A., Chaichit N., Gunnasoot P., Kongsaeree P., Mutikainen I., Roubeau O., Reedijk J., Turpeinen U. (2003). Synthesis, spectroscopic characterization, X-ray crystal structure and magnetic properties of oxalato-bridged copper(II) dinuclear complexes with di-2-pyridylamine. Inorg. Chim. Acta.

[45] Castillo O., Muga I., Luque A., Gutiérrez-Zorrilla J.M., Sertucha J., Vitoria P., Román P. (1999). Synthesis, chemical characterization, X-ray crystal structure and magnetic properties of oxalato-bridged copper(II) binuclear complexes with 2,2′-bipyridine and diethylenetriamine as peripheral ligands. Polyhedron.

[46] Gusev A.N., Nemec I., Herchel R., Bayjyyev E., Nyshchimenko G.A., Alexandrov G.G., Eremenko I.L., Trávníček Z., Hasegawa M., Linert W. (2014). Versatile coordination modes of bis [5-(2-pyridine-2-yl)-1,2,4-triazole-3-yl]alkanes in Cu(ii) complexes. Dalton Trans..

[47] Pokharel U.R., Fronczek F.R., Maverick A.W. (2014). Reduction of carbon dioxide to oxalate by a binuclear copper complex. Nat. Commun..

[48] Boyko A.N., Haukka M., Golenya I.A., Pavlova S.V., Usenko N.I. (2010). [mu]-Oxalato-bis[(2,2’-bipyridyl)copper(II)] bis(perchlorate) dimethylformamide disolvate monohydrate. Acta Cryst..

[49] Castro I., Faus J., Julve M., Mollar M., Monge A., Gutierrez-Puebla E. (1989). Formation in solution, synthesis and crystal structure of μ-oxalatobis[bis(2-pyridylcarbonyl)amido] dicopper(II). Inorg. Chim. Acta.

[50] Li K.-k., Zhang C., Xu W. (2011). [mu]-Oxalato-bis[bis(2,2’-bipyridine)manganese(II)] bis(perchlorate) 2,2’-bipyridine solvate. Acta Cryst..

[51] Kaizaki S., Nakahanada M., Fuyuhiro A., Ikedo-Urade M., Abe Y. (2009). Synthesis, characterization and redox reactivity of l-tartrato bridged dinuclear manganese complex with 2,2′-bipyridine. Inorg. Chim. Acta.

[52] Duan W., Jiao S., Liu X., Chen J., Cao X., Chen Y., Xu W., Cui X., Xu J., Pang G. (2015). Two new supramolecular hybrids based on bi-capped Keggin {PMo_12_V_2_O_42_} clusters and transition metal mixed-organic-ligand complexes. Chem. Res. Chin. Univ..

[53] Shi C., Fan L., Wei P., Li B., Zhang X. (2010). (μ-Oxalato-κ^4^*O*^1^,*O*^2^:*O*^1^’,*O*^2^’)bis[bis(2,2’-bipyridine-κ^2^*N*,*N*’)cobalt(II)] μ_6_-oxido-dodeca-μ_2_-oxido-hexaoxido-hexatungstate(VI). Acta Cryst..

[54] Androš Dubraja L., Jurić M., Torić F., Pajić D. (2017). The influence of metal centres on the exchange interaction in heterometallic complexes with oxalate-bridged cations. Dalton Trans..

[55] Youngme S., Gunnasoot P., Chaichit N., Pakawatchai C. (2004). Dinuclear copper(II) complexes with ferromagnetic and antiferromagnetic interactions mediated by a bridging oxalato group: Structures and magnetic properties of [Cu_2_L_4_(μ-C_2_O_4_)](PF_6_)_2_(H_2_O)_2_ and [Cu_2_L_2_(μ-C_2_O_4_)(NO_3_)_2_((CH_3_)_2_NCOH)_2_] (L = di-2-pyridylamine). Transit. Met. Chem..

[56] Qin C. (2007). μ-Oxalato-κ^2^*O*^1^,*O*^2^:κ^2^*O*^1^’,*O*^2^’-bis[(3,5-dicarboxybenzoato-κ^2^*O*^1^,*O*^1^’)(1,10-phenanthroline-κ^2^*N*,*N*’)copper(II)]. Acta Cryst..

[57] de Faria D.M., Yoshida M.I., Pinheiro C.B., Guedes K.J., Krambrock K., Diniz R., de Oliveira L.F.C., Machado F.C. (2007). Preparation, crystal structures and spectroscopic characterization of oxalate copper(II) complexes containing the nitrogen ligands 4,4′-dimethyl-2,2′-bipyridine and di(2-pyridyl)sulfide. Polyhedron.

[58] Androš L., Jurić M., Planinić P., Žilić D., Rakvin B., Molčanov K. (2010). New mononuclear oxalate complexes of copper(II) with 2D and 3D architectures: Synthesis, crystal structures and spectroscopic characterization. Polyhedron.

[59] Sarma R., Boudalis A.K., Baruah J.B. (2010). Aromatic N-oxide bridged copper(II) coordination polymers: Synthesis, characterization and magnetic properties. Inorg. Chim. Acta.

[60] Stepanian S.G., Reva I.D., Radchenko E.D., Sheina G.G. (1996). Infrared spectra of benzoic acid monomers and dimers in argon matrix. Vib. Spectrosc..

[61] Silverstein R.M., Webster F.X., Kiemle D.J. (2005). Spectrometric Identification of Organic Compounds.

[62] Nakamoto K. (2009). Infrared and Raman Spectra of Inorganic and Coordination Compounds—Part B: Applications in Coordination, Organometallic, and Bioinorganic Chemistry.

[63] Edwards H.G.M., Farwell D.W., Rose S.J., Smith D.N. (1991). Vibrational spectra of copper (II) oxalate dihydrate, CuC2O4·2H20, and dipotassium bis-oxalato copper (II) tetrahydrate, K_2_Cu(C_2_O_4_)_2_·4H_2_O. J. Mol. Struc..

[64] Halaška J., Pevec A., Strauch P., Kozlevčar B., Koman M., Moncol J. (2013). Supramolecular hydrogen-bonding networks constructed from copper(II) chlorobenzoates with nicotinamide: Structure and EPR. Polyhedron.

[65] Godlewska S., Jezierska J., Baranowska K., Augustin E., Dołęga A. (2013). Copper(II) complexes with substituted imidazole and chlorido ligands: X-ray, UV–Vis, magnetic and EPR studies and chemotherapeutic potential. Polyhedron.

[66] Vicente R., Escuer A., Ferretjans J., Stoeckli-Evans H., Solans X., Font-Bardía M. (1997). Structurally alternating copper(II) chains from oxalate and azide bridging ligands: Syntheses and crystal structure of [Cu_2_(µ-ox)(deen)_2_(H_2_O)_2_(ClO_4_)_2_] and [{Cu_2_(µ-N_3_)(µ-ox)(deen)_2_}*_n_*][ClO_4_] n (deen = Et_2_NCH_2_CH_2_NH_2_). J. Chem. Soc. Dalton Trans..

[67] Recio A., Server-Carrió J., Escrivà E., Acerete R., García-Lozano J., Sancho A., Soto L. (2008). Novel Cu(II)-Based Frameworks Built from BIMAM and Oxalate: Syntheses, Structures, and Magnetic Characterizations (BIMAM = Bis(imidazol-yl) methylaminomethane). Cryst. Growth Des..

[68] Halcrow M.A. (2003). Interpreting and controlling the structures of six-coordinate copper(II) centres—When is a compression really a compression?. Dalton Trans..

[69] Yilmaz V.T., Hamamci S., Andac O., Thöne C., Harrison W.T.A. (2003). Mono- and binuclear copper(II) complexes of saccharin with 2-pyridinepropanol synthesis, spectral, thermal and structural characterization. Transit. Met. Chem..

[70] zuah R.T., LR K., Qiu Y., Tregenna-Piggott P.L.W., Brown C.M., Copley J.R.D., Dimeo R.M. (2009). DAVE: A comprehensive software suite for the reduction, visualization, and analysis of low energy neutron spectroscopic data. J. Res. Nat. Inst. Stan..

[71] Bencini A., Gatteschi D. (1990). Electron Paramagnetic Resonance of Exchange Coupled Systems.

[72] Pal P., Konar S., El Fallah M.S., Das K., Bauzá A., Frontera A., Mukhopadhyay S. (2015). Synthesis, crystal structures, magnetic properties and DFT calculations of nitrate and oxalate complexes with 3,5 dimethyl-1-(2′-pyridyl)-pyrazole-Cu(ii). Rsc Adv..

[73] Macrae C.F., Bruno I.J., Chisholm J.A., Edgington P.R., McCabe P., Pidcock E., Rodriguez-Monge L., Taylor R., van de Streek J., Wood P.A. (2008). Mercury CSD 2.0—new features for the visualization and investigation of crystal structures. J. App. Cryst..

[74] Neese F. (2012). The ORCA program system. Wires Comput. Mol. Sci..

[75] Bencini A., Totti F., Daul C.A., Doclo K., Fantucci P., Barone V. (1997). Density Functional Calculations of Magnetic Exchange Interactions in Polynuclear Transition Metal Complexes. Inorg. Chem..

[76] Stoll S., Schweiger A. (2006). EasySpin, a comprehensive software package for spectral simulation and analysis in EPR. J. Magn. Reson..

[77] Kahn O. (1993). Molecular Magnetism.

[78] (2015). Program APEX3.

[79] Sheldrick G. (2015). SHELXT—Integrated space-group and crystal-structure determination. Acta Cryst..

[80] Sheldrick G. (2015). Crystal structure refinement with SHELXL. Acta Cryst..

[81] Sheldrick G. (2008). A short history of SHELX. Acta Cryst..

[82] Farrugia L. (2012). WinGX and ORTEP for Windows: An update. J. App. Cryst..

[83] Adamo C., Barone V. (1999). Toward reliable density functional methods without adjustable parameters: The PBE0 model. J. Chem. Phys..

[84] Weigend F., Ahlrichs R. (2005). Balanced basis sets of split valence, triple zeta valence and quadruple zeta valence quality for H to Rn: Design and assessment of accuracy. Phys. Chem. Phys..

[85] Bencini A., Totti F. (2009). A Few Comments on the Application of Density Functional Theory to the Calculation of the Magnetic Structure of Oligo-Nuclear Transition Metal Clusters. J. Chem. Theory Comput..

[86] Neese F. (2005). Efficient and accurate approximations to the molecular spin-orbit coupling operator and their use in molecular g-tensor calculations. J. Chem. Phys..

