# Supplementary material for: An Oxalate-Bridged Copper(II) Complex Combining Monodentate Benzoate, 2,2′-bipyridine and Aqua Ligands: Synthesis, Crystal Structure and Investigation of Magnetic Properties
Source: Molecules. 2020 Apr 20;25(8):1898. doi: 10.3390/molecules25081898 (PMC7221668; doi:10.3390/molecules25081898)
Supplement: Supplementary file 1 [file molecules-25-01898-s001.pdf]

Electronic Supporting Information

An oxalate-bridged copper(II) complex combining monodentate benzoate, 2,2′-bipyridine and aqua ligands: synthesis, crystal structure and investigation of magnetic properties

Francielli Sousa Santana[a], Matteo Briganti[b], Rafael A. Allão Cassaro[c], Federico Totti[d], Ronny Rocha Ribeiro[b], David L. Hughes[e], Giovana Gioppo Nunes[b], Dayane Mey Reis[a,*]

[a] Departamento de Química e Biologia, Universidade Tecnológica Federal do Paraná, Cidade Industrial, 81280-340 Curitiba, PR, Brazil.

[b] Departamento de Química, Universidade Federal do Paraná, Centro Politécnico, Jardim das Américas, 81531-980 Curitiba, PR, Brazil.

[c] Instituto de Química, Universidade Federal do Rio de Janeiro, 21941-909 Rio de Janeiro, RJ, Brazil.

[d] Dipartimento di Chimica "U.Schiff" and UdR INSTM Università degli Studi di Firenze, Via della Lastruccia 3-13, 50019 Sesto Fiorentino, Italy.

[e] School of Chemistry, University of East Anglia, Norwich NR4 7TJ, United Kingdom.

* Corresponding author.

E-mail address: dayane@utfpr.edu.br (Dayane Mey Reis).

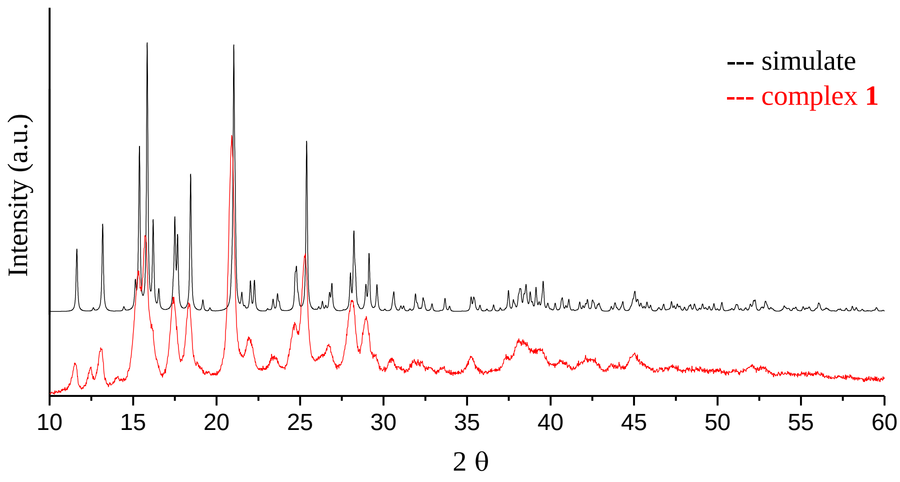

Figure S1: Comparison between simulated and experimental PXRD patterns of complex **1** (scan velocity: 0.005 °s^{-1}.

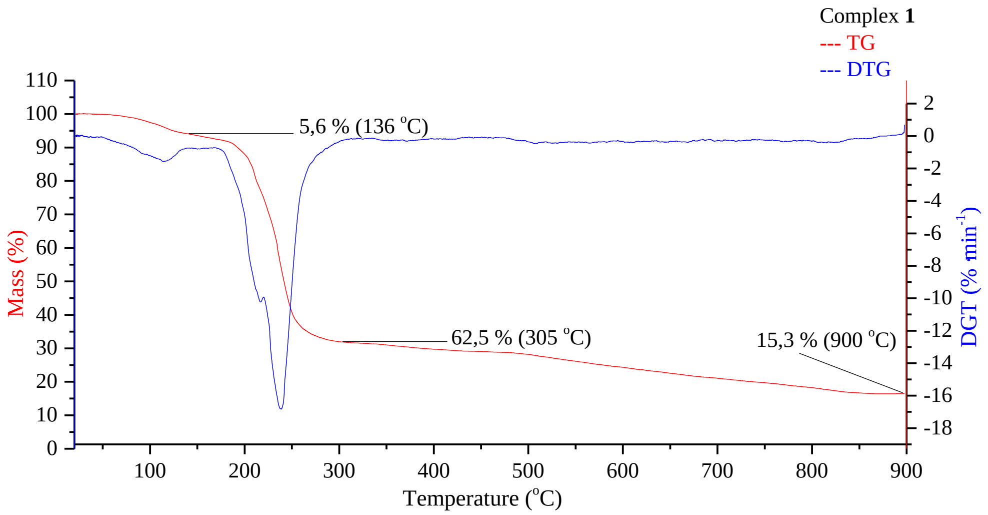

Figure S2: Thermogravimetric analysis (TG and DTG) profiles obtained for **1** in O_2/N_2 as carrier gas and temperature range of 20 to 900 °C.

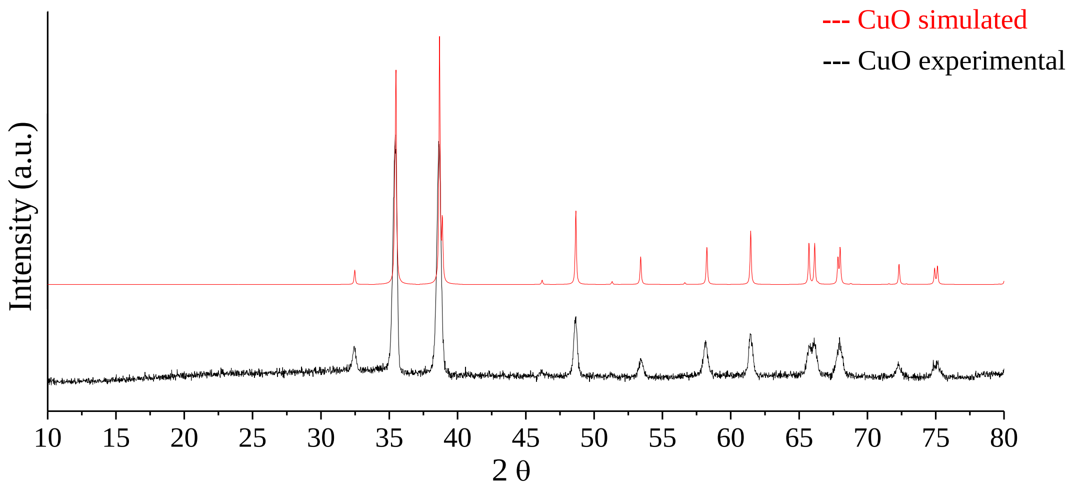

Figure S3: Comparison between simulated and experimental PXRD patterns of CuO (scan velocity: 0.005° s^{-1}).

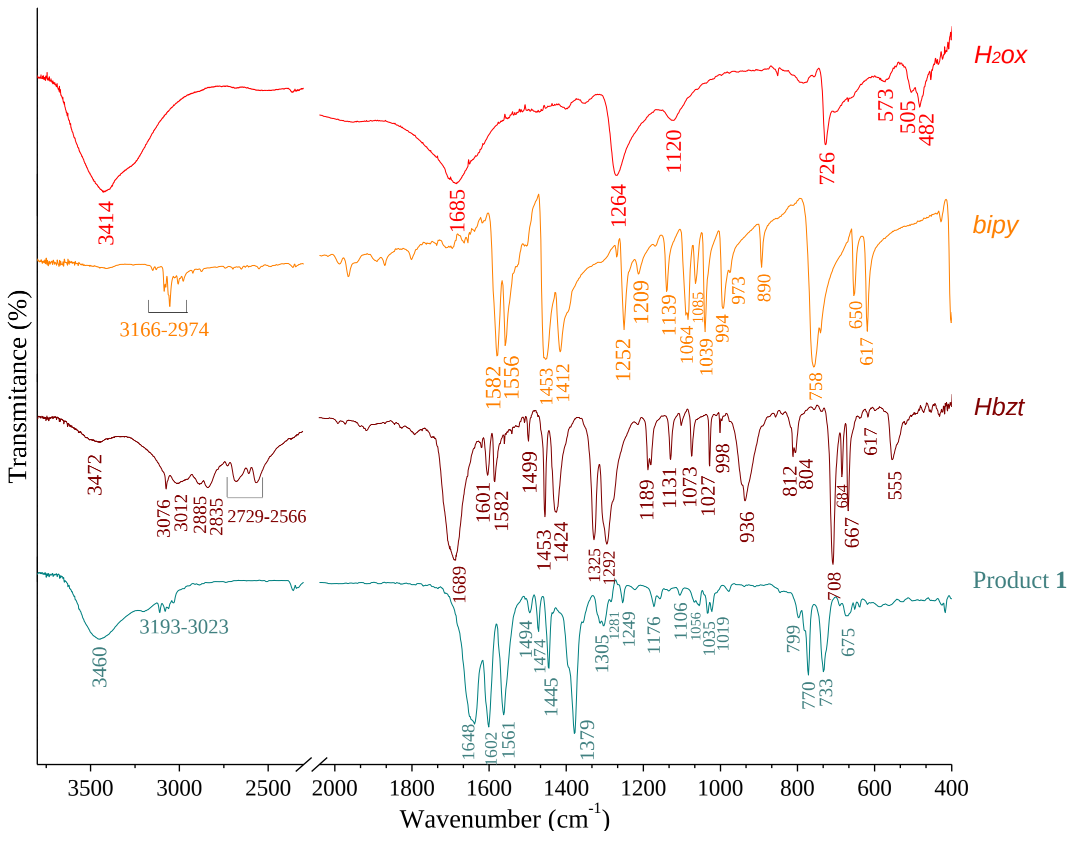

Figure S4: Infrared spectra of **1** and its starting materials, H_2*ox*·$2H_2O$, H*bzt*, and *bipy* recorded from KBr pellets.

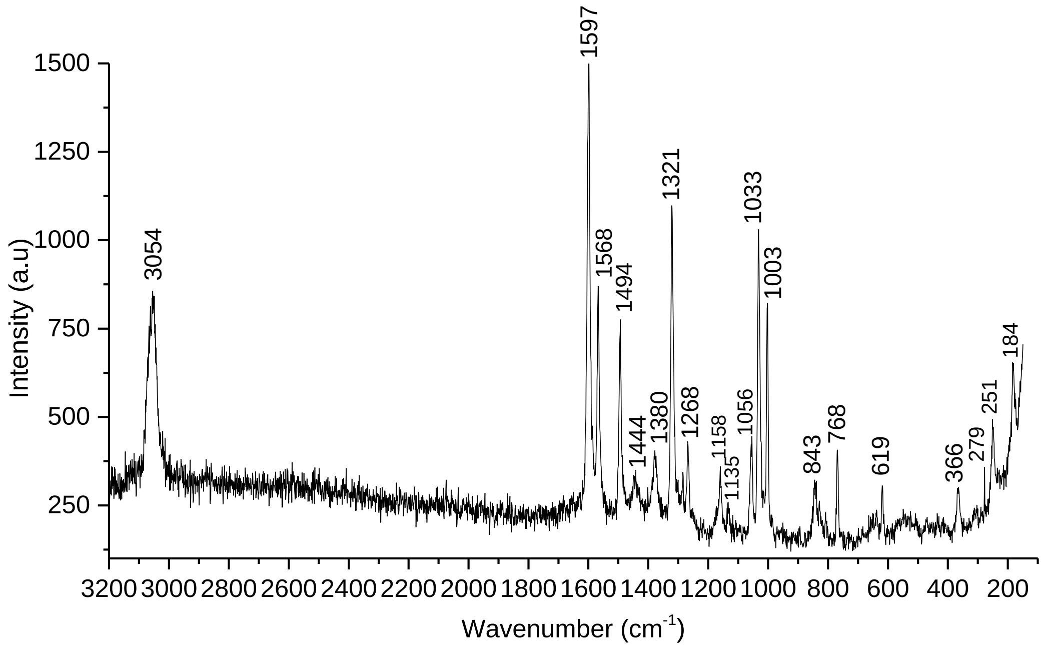

Figure S5: Raman spectrum recorded for **1** with exposure time of 10 s and power at 50 % (laser: He-Ne, 632.8 nm).

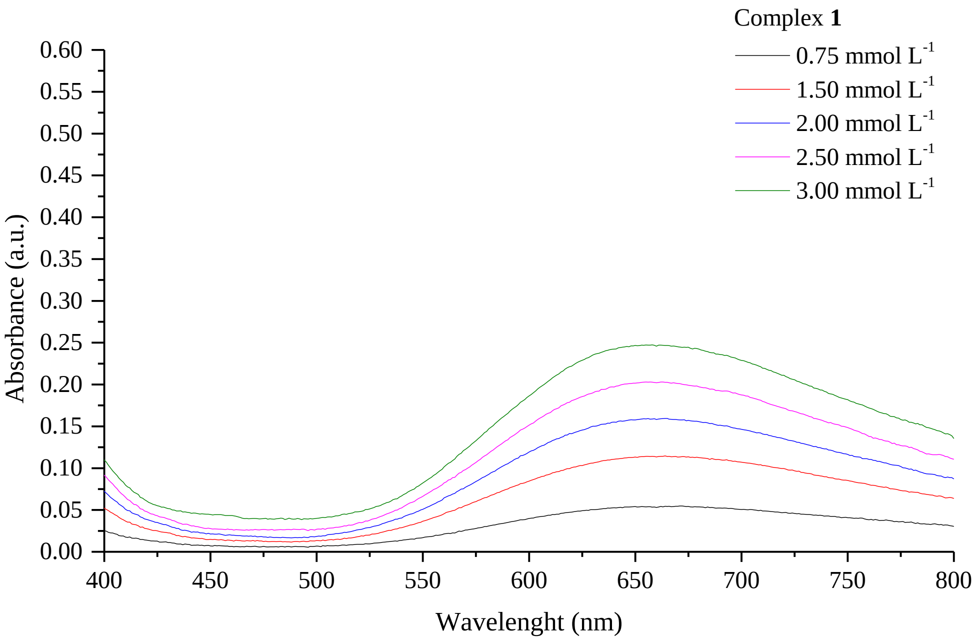

Figure S6: UV-Vis absorption spectra recorded for complex **1** at 3.00, 2.50, 2.00, 1.50, and 0.75 mmol L^{-1} in water.

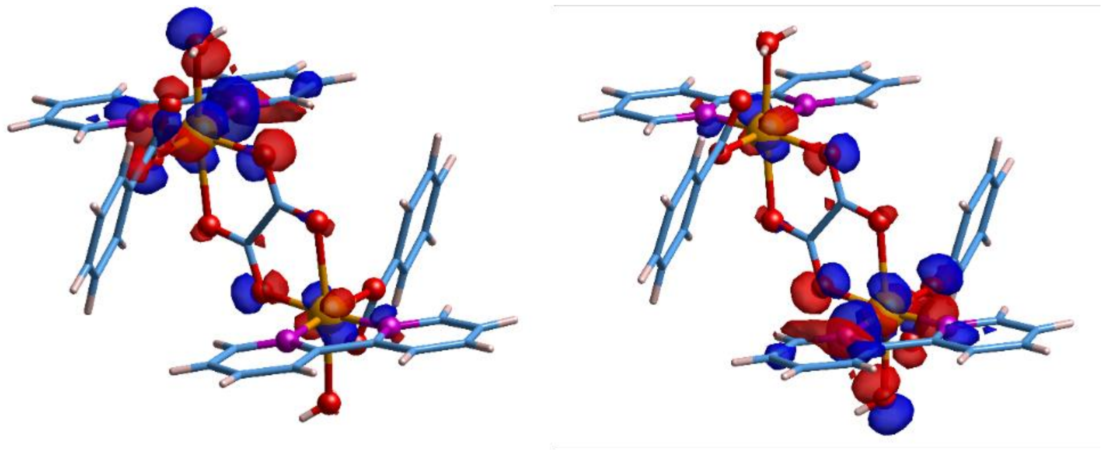

Figure S7: Graphic representation of the two occupied broken symmetry (BS), spin up and spin down, magnetic orbitals for complex **1.** The electron density isovalue was set to 0.04 $e/(a_0)^3$. Light-blue: carbon, white: hydrogen; red: oxygen; orange: copper; pink: nitrogen.

Table S1: Values of 2θ angles for the experimental and simulated PXRD diffractograms of **1** from 10 to 30° displaying a difference of *ca* 0.1°

**Experimental (2θ)**	**Simulated (2θ)**
11.58	11.66
12.5	12.58
13.06	13.16
14.08	14.46
15.32	15.34
15.7	15.08
16.14	16.18
np*	16.56
17.38	17.5
18.32	18.42
**	19.18
20.9	21.06
np	21.52
21.96	22.18
23.42	23.6
24.66	24.72
25.26	25.38
**	26.3
26.72	26.88
28.08	28.18
28.98	29.12
29.46	29.6

*np = not present (it is a low-intensity peak in the theoretical diffractogram).
** = there is a shoulder at the nearby experimental peak that could refer to this low-intensity peak present in the simulated diffractogram.

Table S2: Hydrogen bond parameters (Å, °)

D–H···A	D–H	H···A	D···A	D–H···A
C(4)–H(4)···O(8)^{ii}	0.92	2.28	3.096(4)	143.9
O(1)–H(1B)···O(8)^{iii}	0.817(18)	2.21(2)	2.954(3)	152(3)
O(1)–H(1A)···O(7)	0.836(18)	1.95(2)	2.723(3)	153(3)

Symmetry codes: (ii) -x+1, -y+1, -z+1; (iii) x-1, y, z.

Table S3: Thermal data for **1**.

Step	Temp. range (°C)	Mass loss (%)		Possible moities lost through decomposition
		Found	Calculated	
**I**	54.8-135.8	5.6	4.5	2 H_2O
**II**	135.8-305.2	62.5	68.9	2 *bzt* + 2 *bipy*
**III**	305.2-900	15.3	10.9	*ox*
**Total**	**-**	**83.4**	**84.3**	

Table S4: Tentative assignments for the IR (cm^{-1}) spectrum recorded for complex **1** [1-4]

Tentative assignments^{a}	Complex 1	*H₂ox*	*Hbzt*	*bipy*
$\nu_{as,s}$(OH)	3460	3414	3472-2835	-
ν_{as}(CH)	3193-3023	-	3076-3012	3166-2974
ν_{s}(CH)	-	-	2885-2566	-
$\nu_{as}(CO)_{COOH}$	-	1685	1689	-
$\nu_{as}(CO)_{COO^-}$	1648	-	-	-
ν_{s}(CC e CN)	1602-1412	-	1601-1453	1582-1412
$\nu_{s}(CO)_{COO^-}$	1379	-	-	-
δ(CH)	1305-1019	1120; 726	1424-998	1252-973
β(CH)	779-675	-	812-555	890-617
β(CC)	-	726	-	-
$\beta(OH)_{COOH}$	-	-	936	-
$\delta(OH)_{COOH}$	-	1264	1424	-

(a) ν_s, asymmetrical stretching; ν_s, symmetrical stretching; δ, in-plane angular deformation; β, out-of-plane angular deformation.

Table S5: Tentative assignments for the Raman scattering (cm^{-1}) spectrum recorded for complex **1** [5-8]

Tentative assignments [(a)]	Complex 1
ν_s(OH)	3054
$\nu_{ass}(CO)_{COO^-}$	1597
ν_s(CC ou CN)	1597-1494
δ(CH)	1444
$\nu_s(CO)_{COO^-}$	1380
δ(CCH)	1268
$\delta(CC)_{ring}$	1056
δ(OCO)	843
ν(Cu-O, Cu-N)	619-366

[(a)] ν_{as}, asymmetrical stretching; ν_s, symmetrical stretching; δ, in-plane angular deformation.

References

1. Silverstein, R. M.; Webster, F. X.; Kiemle, D. J. *Spectrometric identification of organic compounds*. 7ed.; John Wiley & Sons: 2014.
2. NAKAMOTO, K., *Infrared and Raman Spectra of Inorganic and Coordination Compounds - Part B: applications in coordination, organometallic, and bioinorganic chemistry.* 6 ed.; Wiley & Sons: New Jersey, 2009.
3. Stepanian, S. G.; Reva, I. D.; Radchenko, E. D.; Sheina, G. G., Infrared spectra of benzoic acid monomers and dimers in argon matrix. *Vib. Spectrosc.* **1996,** 11, 123-133.
4. Androš, L.; Jurić, M.; Planinić, P.; Žilić, D.; Rakvin, B.; Molčanov, K., New mononuclear oxalate complexes of copper(II) with 2D and 3D architectures: Synthesis, crystal structures and spectroscopic characterization. *Polyhedron* **2010,** 29, 1291-1298.
5. Helios, K.; Wysokiński, R.; Pietraszko, A.; Michalska, D., Vibrational spectra and reinvestigation of the crystal structure of a polymeric copper(II)–orotate complex, $[Cu(\mu\text{-}HOr)(H_2O)_2]_n$: The performance of new DFT methods, M06 and M05-2X, in theoretical studies. *Vib. Spectrosc.* **2011,** 55, 207-215.
6. Edwards, H. G. M.; Farwell, D. W.; Rose, S. J.; Smith, D. N., Vibrational spectra of copper (II) oxalate dihydrate, $CuC_2O_4{\cdot}2H_2O$, and dipotassium bis-oxalato copper(II) tetrahydrate, $K_2Cu(C_2O_4)_2{\cdot}4H_2O$. *J. Mol. Struct.* **1991,** 249, 233-243.
7. Wang, Z. P.; Tang, X. D.; Ding, Z. J., Raman and photoluminescence spectroscopy study of benzoic acid at high pressures. *J. Phys. Chem. Solids.* **2005,** 66, 895-901.
8. Brolo, A. G.; Jiang, Z.; Irish, D. E., The orientation of 2,2′-bipyridine adsorbed at a SERS-active Au(111) electrode surface. *J. Electroanal. Chem.* **2003,** 547, 163-172.
